# Transcriptomic analysis of Streptococcus pneumoniae serotype 1 reveals serotype-specific gene regulation

**DOI:** 10.1099/mgen.0.001582

**Published:** 2025-12-05

**Authors:** Pisut Pongchaikul, Karsten Hokamp, Morten Kjos, Chrispin Chaguza, Teerawit Audshasai, Stavros Panagiotou, Reham Yahya, Laura Bricio-Moreno, Jay C. D. Hinton, Aras Kadioglu, Marie O’Brien

**Affiliations:** 1 Ramathibodi Medical School, Faculty of Medicine Ramathibodi Hospital, Mahidol University, Bang Phli, Samut Prakan 10540, Thailand; 2 Integrative Computational BioScience Center, Mahidol University, Nakhon Pathom 73170, Thailand; 3 Department of Clinical Infection Microbiology and Immunology, Institute of Infection Veterinary and Ecological Sciences, University of Liverpool, Liverpool, UK; 4 Department of Genetics, School of Genetics and Microbiology, Smurfit Institute of Genetics, Trinity College, Dublin, Ireland; 5 Faculty of Chemistry, Biotechnology and Food Science, Norwegian University of Life Sciences, Ås, Norway; 6 Department of Host-Microbe Interactions, St Jude Children's Research Hospital, Memphis, Tennessee, USA; 7 Department of Microbiology, Faculty of Pharmacy, Mahidol University, Bangkok, Thailand; 8 Division of Medical Education, School of Medical Sciences, Faculty of Biology Medicine and Health, University of Manchester, Manchester, UK

**Keywords:** bacterial competence, pneumococcal transcriptomics, serotype 1 hypervirulence, *Streptococcus pneumoniae*

## Abstract

*Streptococcus pneumoniae* (*Sp*) is an opportunistic pathogen that colonizes the mucosal surfaces of the human upper respiratory tract. While transcriptomic studies of *Sp* have become more common, most have focused on laboratory-adapted strains such as D39 or TIGR4. These strains, though widely used in research, may not fully capture the biology of clinical isolates, particularly the hypervirulent serotype 1 (S1). S1 is clinically significant due to its association with invasive disease, epidemic outbreaks and a distinct global distribution, particularly in regions with a high pneumococcal disease burden. Unlike many other serotypes, S1 is frequently linked to hypervirulence and a propensity for rapid spread, making it a high-priority target for understanding the molecular mechanisms underpinning pneumococcal pathogenesis. In this study, we conducted a comprehensive *in vitro* transcriptomic analyses of *Sp* S1 strains, positioning this work as a valuable resource for the pneumococcal research community. Using a straightforward approach, we cultured three distinct S1 strains – ST306, ST217 and ST615, representing European, African and South American S1 lineages, respectively – in Brain Heart Infusion medium and compared transcriptomic profiles during exponential growth to those of the well-characterized laboratory-adapted D39 strain. Our analysis revealed significant differential expression of 292 genes in all three S1 isolates compared to D39. Among these, 151 genes had higher expression, including those involved in competence pathways and purine metabolism, while 141 genes exhibited lower expression, particularly those linked to lactose metabolism and iron/amino acid transport. These findings underscore the distinct molecular features of S1 strains, which likely contribute to the unique pathogenic properties of this serotype. The identification of the distinct transcriptional signatures of hypervirulent S1 strains paves the way for future efforts to design targeted therapeutics against pneumococcal S1 infections.

**Impact Statement**

Our study presents the *in vitro* comparative transcriptomic analysis of geographically diverse *Streptococcus pneumoniae* serotype 1 isolates, a hypervirulent lineage underrepresented in bacterial gene expression profiling. By defining core serotype-specific transcriptional signatures and providing an open-access exploration tool (StrepCom), this work addresses a critical gap in pneumococcal transcriptomics and offers a valuable resource to inform future mechanistic and translational studies, including *in vivo* investigations.

## Data Summary

The datasets generated for this study have been deposited at Gene Expression Omnibus (accession nos. GSE279694 and GSE159305).

## Introduction

*Streptococcus pneumoniae* (*Sp*) (or the pneumococcus) is a common commensal of the human nasopharynx but also a devastating pathogen. Invasive pneumococcal diseases (IPDs) such as sepsis, pneumonia and meningitis are major causes of morbidity and mortality in Europe as well as globally, with the highest burden of disease found in young children and the elderly [1,2]. Of the more than 100 pneumococcal serotypes [3] identified so far, serotype 1 (S1) pneumococci count amongst the most prevalent strains causing invasive diseases, especially among vulnerable populations in poor resource settings [4] with a greater burden of comorbidities. Intriguingly, despite the introduction of pneumococcal conjugate vaccination, the incidence of IPDs caused by S1 is persisting [5,6], hence the necessity to further understand the biology of S1 pneumococci. We previously reported the *in vitro* and *in vivo* phenotypes of S1 pneumococci, specifically on ST217, ST306 and ST615 clones which are representative of the geographical cluster lineage A (Europe), B (Africa) and C (South America), respectively. Notably, the ST217 was shown to produce large quantities of pneumolysin, due to a more rapid autolysis compared to other serotypes [7]. Using murine models, we subsequently showed that, contrary to current assumptions, the African ST217 is readily carried in the nasopharynx, but at a lower density and for a shorter duration than is typical for other pneumococcal serotypes [8] recapitulating the findings from pneumococcal carriage surveys in humans [9,10]. We also made the unexpected finding that ST615 harbours the expression of two haemolytic variants of pneumolysin (Ply), i.e. a cell-wall restricted fully haemolytic Ply, and a non-haemolytic cytosolic pool – a phenotype never before reported in the pneumococcus [11].

Defined as a high attack rate pathogen, S1 pneumococci are believed to present the ability to rapidly transition from the nasopharynx to deeper tissues [7,12]. As such, understanding the expression patterns associated with the growth of hypervirulent strains such as S1 pneumococci may help develop more effective preventative and therapeutic approaches. We and others have previously used RNA-sequencing (RNA-seq) approaches to correlate functional traits and gene expression data. Aprianto *et al*. were the first to describe a dual-transcriptomic analysis of early pneumococcal infection in a time-resolved manner [13]. Minhas *et al*. used dual RNA-seq to simultaneously quantify genome-wide transcriptional responses of both host and pathogen in serotype 14 pneumococcus-infected mice [14]. They showed that one SNP in pneumococci could induce significantly diverging host responses that, in turn, determined the outcome of infection. Transcriptomic studies were also conducted to investigate interactions between serotype 19F pneumococci and viruses, e.g. influenza virus A [15]. One of the most comprehensive analyses was reported for the serotype 2 strain D39, whereby the transcriptome was assessed by RNA-seq in 22 different conditions relevant to infection and mimicking various tissue niches, mode of transmission, temperatures and genetic transformation [16]. These investigations led to the creation of a mineable transcriptome database for D39 pneumococci. RNA-seq also underpinned the discovery that host haemoglobin can stimulate the growth of D39 pneumococci and act as a cue for the host environment [17]. Other studies used RNA-seq to investigate the effect of e-cigarette vapour on the transcriptome and virulence properties of TIGR4 [18]. Interestingly, however, the majority of these studies were mainly conducted in laboratory-adapted strains such as D39 or TIGR4. Beyond datasets centred on D39/TIGR4, recent work with clinical isolates and host-like conditions has expanded the pneumococcal transcriptome landscape. Ramos-Sevillano *et al*. [19] carried out cultures in human serum and cerebrospinal fluid (CSF) combined with RNA-seq and metabolomics to define metabolic circuitry underpinning *Sp* systemic virulence (6B BHN418 vs. D39). Complementing this, Oh *et al*. [20] performed time-resolved *in vivo* RNA-seq during pneumonia-derived sepsis in murine models, while Chong *et al*. [21] contrasted *in vivo* vs. *in vitro* competence gene expression dynamics. Finally, direct bacterial RNA-seq from patient CSF has recovered transporter and stress-response programmes active during human *Sp* meningitis [22], and *ex vivo* human plasma/CSF studies similarly showed rapid remodelling of pneumococcal transcription [23].

To persist as a commensal bacterium and a pathogen in its sole host, i.e. humans, *Sp* must subsist in different tissue niches with differential cellular, nutritional and microbiological environments. In the present study, we sought to define the most fundamental transcriptomic differences between pneumococcal S1 and D39 and asked whether and how *Sp* S1 differs in its gene expression compared to the laboratory strain D39 during exponential growth phases *in vitro*. While transcriptomic data were collected across five growth stages [early exponential phase (EEP), mid-exponential phase (MEP) and late exponential phase (LEP); early stationary phase (ESP) and late stationary phase (LSP)], the primary focus of our analysis was on the exponential growth phases, as they represent the period of active bacterial proliferation that is most relevant to dissemination and invasion during infection. Our study provides a resource for the pneumococcal research community with detailed transcriptomic data for clinically relevant S1 strains, supporting future research aimed at understanding and combating pneumococcal disease.

## Methods

### Bacterial strains

The *Sp* strains used in this study are designated by their sequence type (ST) followed by serotype (S), as follows: ST306/S1, ST615/S1, ST217/S1 and D39/S2. The strain ST306 was kindly provided by Dr Lucy Hathaway, Institute for Infectious Diseases, University of Bern, Switzerland, while the strain ST615 was provided by Prof Francois Trottein, at the Pasteur Institute in Lille, France. The strain ST217 is a blood isolate from a patient presenting with septicaemia in Blantyre, Malawi [8]. Strain D39 is the laboratory-adapted strain NCTC7466 [24].

### Growth curve

The four target strains of *Sp* were inoculated into 50 ml of Brain Heart Infusion (BHI) broth (Oxoid, UK) at a starting inoculum of ~100 c.f.u. per culture tube and cultured at 37 °C. Bacterial growth was monitored by measuring the optical density at a wavelength of 600 nm (OD600) using a microplate reader (Thermo Helios Alpha, Thermo Scientific, USA). Three independent experiments were performed for each isolate and the results were averaged. Cultures were harvested at five distinct growth phases – EEP, MEP, LEP, ESP and LSP – as defined in our previous studies [25]. The growth rate was calculated based on the following equation:


$$Nt=\frac{K}{1+(\frac{K-N0}{N0})e^{-rt}}$$

Where:

*N*0 is the population size at the beginning of the growth curve

*K* is the maximum possible population size in a particular environment, or the carrying capacity.

*r* is the growth rate that would occur if there were no restrictions imposed on total population size.

*t* is the time used in the growth curve.

The calculation was performed using the ‘growthcurver’ package, implemented in R.

### RNA extraction

RNA extraction was performed using a phase separation method with TRIzol® (Invitrogen, UK). Briefly, cultures were centrifuged and resuspended in TRIzol® for cell lysis, followed by chloroform extraction to separate the RNA into the aqueous phase. The resulting RNA fraction was precipitated with ethanol and resuspended in DNase/RNase-free water (ThermoFisher Scientific, UK) to prevent RNA degradation. RNA sequencing was performed by Vertis Biotechnologie AG (Freising, Germany) on an Illumina NextSeq 500 platform (Illumina, San Diego, CA, USA). The rRNA molecules were depleted from the RNA samples using a mix of Ribo-Zero rRNA Removal Kits from Epicenter (human/mouse kit and bacteria kit in a 1 : 1 ratio). In a prior RNA-seq study carried out by our group using the same extraction method, sequencing platform and analysis pipeline, the RNA-seq results were shown to be consistent with quantitative reverse transcriptase polymerase chain reaction (RT-PCR) validation [8]. Although qRT-PCR validation was not repeated in the present study, the methodological consistency provides confidence that the current dataset is of comparable quality.

### Transcriptomic analysis

The quality assessment of raw reads was conducted using FastQC [26]. Homologous genes of all four strains were identified using Panaroo version 1.5.0 [27] with the settings ‘--clean-mode sensitive --refind-mode off --remove-invalid-genes’. Only clusters with 1 : 1 homologue assignment amongst the four strains were kept. Sequence information for each strain was derived from GenBank entries with the following accession numbers: CP000410 (D39), CP071871 (ST217), FQ312039 (ST306) and NZ_LN831051 (ST615). The mapping of the reads against the reference genomes was carried out through bowtie2 version 2.4.2 [28] with the flag ‘--very-sensitive-local' to apply soft-trimming and detect as many matches as possible. Any hits with an XS:i tag (indicating detection of additional alignments for a read) were excluded to focus only on uniquely mapped reads. A filter on expression levels was applied to include only genes that had a minimum transcript count per million (TPM) of 10 in more than half of the samples from each contrast group (S1 and S2). Reads were aggregated into a read-count matrix through featureCounts version 2.0.3 [29] in stranded mode.

Principal component and differential expression analyses were performed using the DESeq2 package (version 1.40.2) [30] in R with the Benjamini-Hochberg method for multiple-testing correction. Fold changes and p-values were calculated based on the comparison of three D39 samples (EEP, MEP and LEP) against nine S1 samples (EEP, MEP, LEP x ST306, ST217 and ST615). Genes were filtered by adjusted *P*-value <0.05 and |log2 fold change (log2FC)| ≥ 1.00. The functional categories of differentially expressed genes (DEGs) were subsequently predicted using blastp against NCBI’s Clusters of Orthologous Groups (COGs) 2020 database. The analysis of protein–protein interaction was performed in *silico* using STRING version 12.0, a web-based prediction platform [31]. The principal component analysis (PCA) plots were generated in the plotly package version 4.10.3 [32], and the volcano plots were made using the Bioconductor package EnhancedVolcano version 1.24 [33].

### Community data resource: StrepCom online visualization tool

An online browser called StrepCom for interactive viewing of expression values, fold changes and annotation information was set up at https://bioinf.gen.tcd.ie/cgi-bin/strepcom.pl. It was based on SalCom, the *Salmonella enterica* serovar *Typhimurium* Gene Expression Compendium [25] developed by Karsten Hokamp (Trinity College Dublin) and Jay Hinton (University of Liverpool).

## Results

### Planktonic growth dynamics of *Sp* strains in BHI medium

**Fig. 1.**
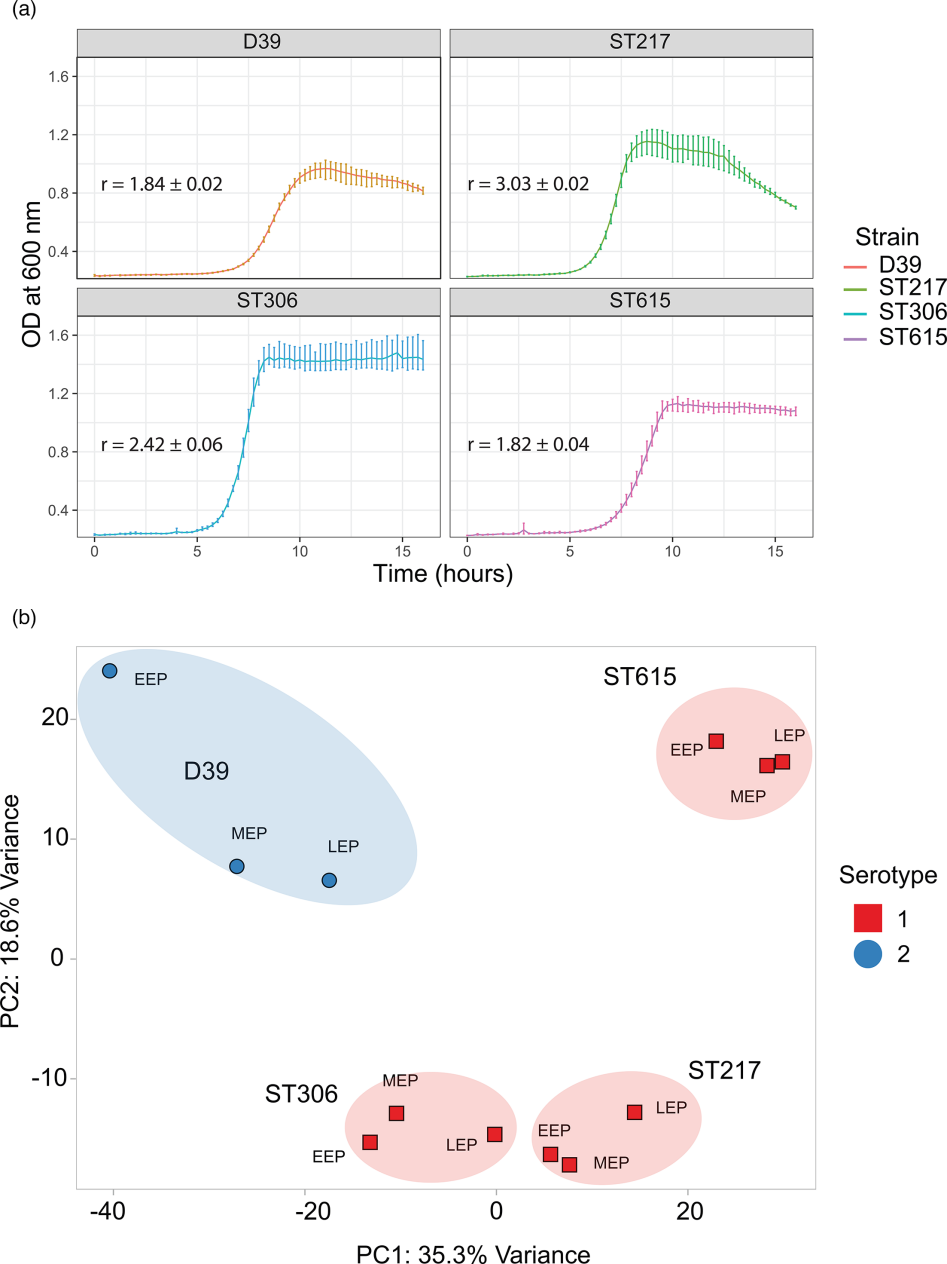
Planktonic growth curves and PCA analysis. (**a**) Planktonic growth curves of *Sp* strains in BHI medium. Growth dynamics of four *Sp* strains – three S1 clinical isolates (ST217, ST306 and ST615) representing African, European and South American lineages, respectively, and the serotype 2 laboratory reference strain D39 – were monitored over 16 h in BHI broth at 37 °C. The x-axis represents time in hours and the y-axis represents OD measured at 600 nm (OD600). Each curve represents the average of three independent experiments, with error bars indicating variability between replicates. For transcriptomic analyses, bacterial cultures were harvested at five defined growth stages: EEP, MEP, LEP, ESP and LSP. These growth phases provided the biological framework for subsequent RNA-seq profiling to examine phase-specific transcriptional differences between S1 strains and the D39 reference. r, growth rate. The growth rate of each strain was shown in each plot (*r*±sem) (**b**) PCA of the RNA-seq data showing the global transcriptional relationships among the four *Sp* strains (D39, ST217, ST306 and ST615). Each point represents one biological replicate, and colours denote strain identity. Samples were collected at five distinct growth stages (EEP, MEP, LEP, ESP and LSP), indicated by shape coding. The PCA plot highlights both the clustering of replicates within each strain and the separation between strains, reflecting strain-specific transcriptional signatures across the growth curve. Strain identity was the primary driver of transcriptome variation, with growth phase contributing a secondary axis.

All four strains of *Sp* were grown in BHI broth medium, and absorbance readings were plotted against time to generate bacterial growth curves (Fig. 1a). On comparing the planktonic growth of the four strains, the highest OD600 was reached by ST306/S1, followed by ST217/S1, ST615/S1 and D39/S2. The growth curves of ST217/S1 (growth rate, *r*=3.03**±**0.02) and ST306/S1 (*r*=2.42**±**0.06) both reached a plateau at 8 h with a maximum OD600 of 1.2 and 1.5, respectively. ST306/S1 showed a stable absorbance reading up to 16 h, while OD600 for ST217/S1 declined rapidly at 12.5 h. This decline is consistent with prior reports of rapid autolysis for ST217/S1 [7]. In contrast, ST615/S1 and D39/S2 showed slower growth rates (*r*=1.82**±**0.04 and 1.84**±**0.02), reaching a maximum OD600 of ~1.2 and 1.0, respectively, with a plateau (i.e. stationary phase) starting at 10 h (ST615/S1) and 11 h (D39/S2). The exponential growth phase was segmented into three intervals, i.e. EEP, mid MEP and LEP exponential phase, and similarly, the stationary growth phase was segmented into ESP and LSP. Within each interval and for each of the four strains, cultures were collected for mRNA extraction and transcriptomic analysis.

### Transcriptomic profiling reveals phase-specific gene expression in *Sp*

We hypothesized that the transcriptomic profiles of pneumococcal planktonic cultures may provide insight into the molecular basis of the virulence of *Sp* S1 strains. A total number of 14,505,831 to 44,567,392 reads was generated from the cDNA library prepared from the RNA obtained from the four pneumococcal strains (Table S1, available in the online Supplementary Material). The overall transcriptomic profiles of all four strains were interpreted using PCA and determined that the first component (PC1) and the second component (PC2) were responsible for 34.8% and 17.6% of the variance, respectively (Fig. 1b). To capture the full transcriptional dynamics, samples were collected at five distinct growth stages (EEP, MEP, LEP, ESP and LSP). The PCA demonstrated that the exponential growth phase samples (EEP, MEP and LEP) clustered tightly within each strain, confirming low intra-phase variability (Figs 1b and S1). While the growth phase accounted for a secondary axis of variation, the primary separation was strain-specific, supporting our rationale for grouping the exponential phases in downstream analyses to highlight conserved differences between S1 isolates and D39 (Fig. S1). This decision reflects the biological relevance of exponential growth to the actively proliferating state of pneumococci during infection, while stationary phase transcriptomes are provided as part of the dataset for future community use.

### Global transcriptional differences highlight metabolic divergence

**Fig. 2.**
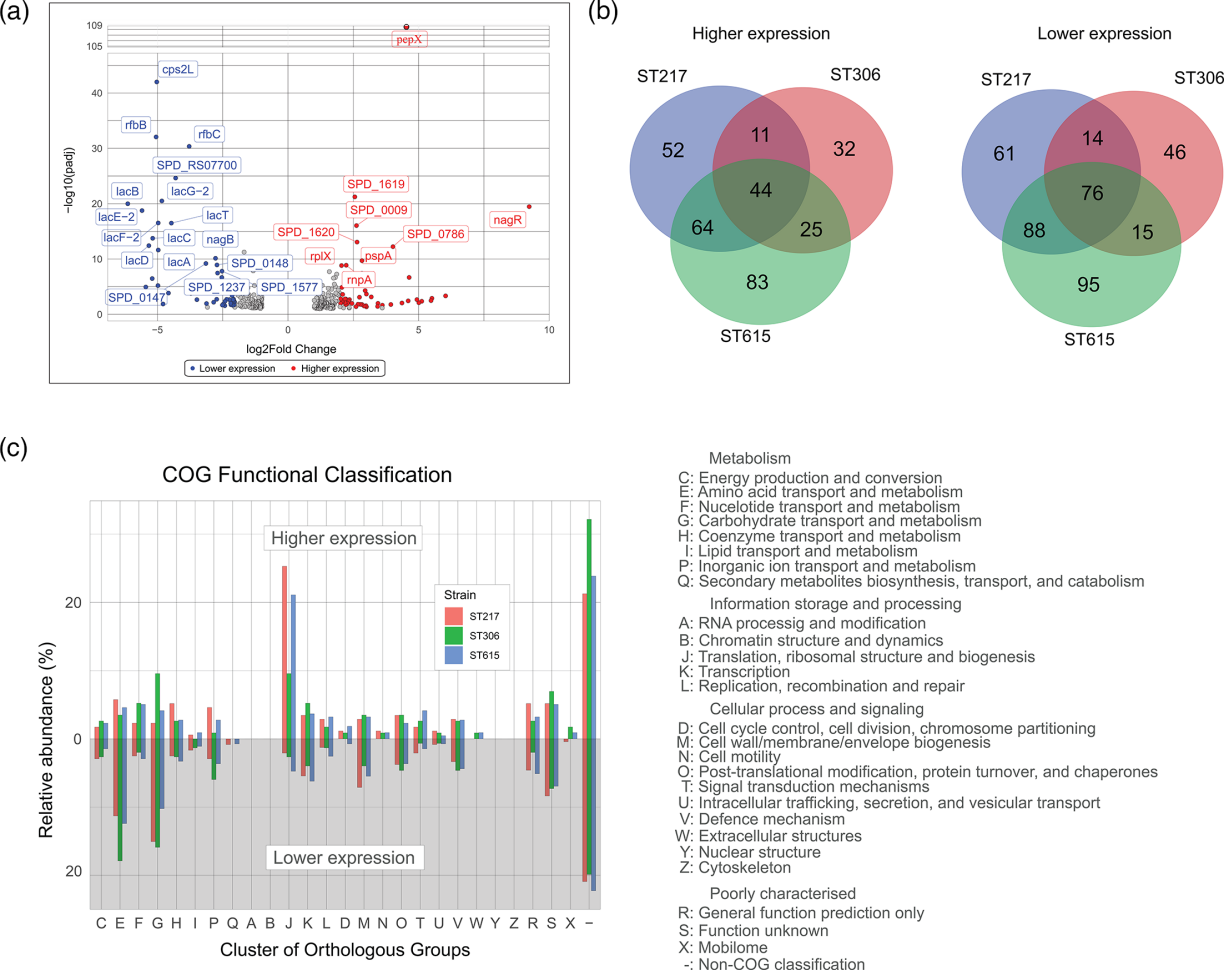
Overview of the transcriptomic expression of pneumococcal serotype 2 (D39) and S1 (ST615, ST615 and ST217). (**a**) Volcano plot showing DEGs identified during the exponential growth of S1 strains compared with D39. The x-axis shows the log2FC and the y-axis shows the -log10(adjusted *P*-value). Genes were considered significantly differentially expressed if they met the cut-off thresholds of |log2FC|≥1.0 and adjusted *P*-value (Benjamini–Hochberg correction) <0.05, as defined in DESeq2 analysis. Red points represent significantly higher-expressed genes in S1 strains relative to D39, while blue points represent significantly lower-expressed genes. Grey points represent genes not meeting the threshold. The volcano plot illustrates that many of the most highly expressed genes in S1 belonged to the competence system (e.g. *comCDE* and associated regulons), purine metabolism and ribosomal protein genes, while the most strongly lower-expressed genes were concentrated in carbohydrate transport systems, especially the lactose operons (*lacABCD* and *lacTFEG*) and selected amino acid/metal transporters. (**b**) Venn diagrams displaying the distribution of DEGs across individual S1 strains compared with D39. The left panel shows DEGs with higher expression relative to D39, and the right panel shows those with lower expression. Overlapping regions indicate genes consistently differentially expressed across all three S1 strains, while non-overlapping regions reflect strain-specific changes. This analysis demonstrates a substantial core set of transcriptional differences shared among the S1 strains, suggesting conserved S1-specific regulatory features, alongside subsets of genes with divergent regulation reflecting lineage-specific adaptations. (**c**) Stacked bar charts of COG categories assigned to the DEGs. Functional categories were determined using the COG classification system. The upper panel depicts categories of genes higher expressed in S1 relative to D39, including translation and ribosomal structure, nucleotide metabolism (purine/pyrimidine biosynthesis) and competence/regulatory functions. The lower panel depicts categories of lower expressed genes, dominated by carbohydrate transport and metabolism (including lactose and other sugar transport operons), amino acid transport and metabolism and inorganic ion transport (e.g. iron and manganese acquisition systems). The order of COG categories is indicated on the right side of each bar. Together, the volcano plots, Venn diagrams and COG assignments illustrate both the conserved and strain-specific transcriptional signatures that distinguish clinical S1 isolates from the laboratory strain D39.

We next sought to compare the expression patterns of *Sp* S1 (i.e. ST615, ST217 and ST306) to that of the orthologous genes in *Sp* D39 during the exponential phase of their planktonic growth. The cut-off threshold used to determine differential expression was set at |log_2_FC|≥1.00 and adjusted *P*-value <0.05. The volcano plot (Fig. 2a) showing the log2FC plotted against the -log10(adjusted *P*-value) highlights the DEGs common to all S1 strains compared to D39. Overall, there were 292 DEGs (151 genes were more highly expressed in all S1 strains and 141 genes displayed lower expression (Fig. 2b). In ST615, a total of 488 genes were differentially expressed compared to D39 : 216 genes had higher expression levels, and 274 genes showed lower expression levels. In ST217, 410 genes were differentially expressed: the expression of 171 genes was higher, while another 239 genes had lower expression levels. In ST306, 263 genes were differentially expressed: 112 genes had higher expression, and 151 genes were lower.

**Fig. 3.**
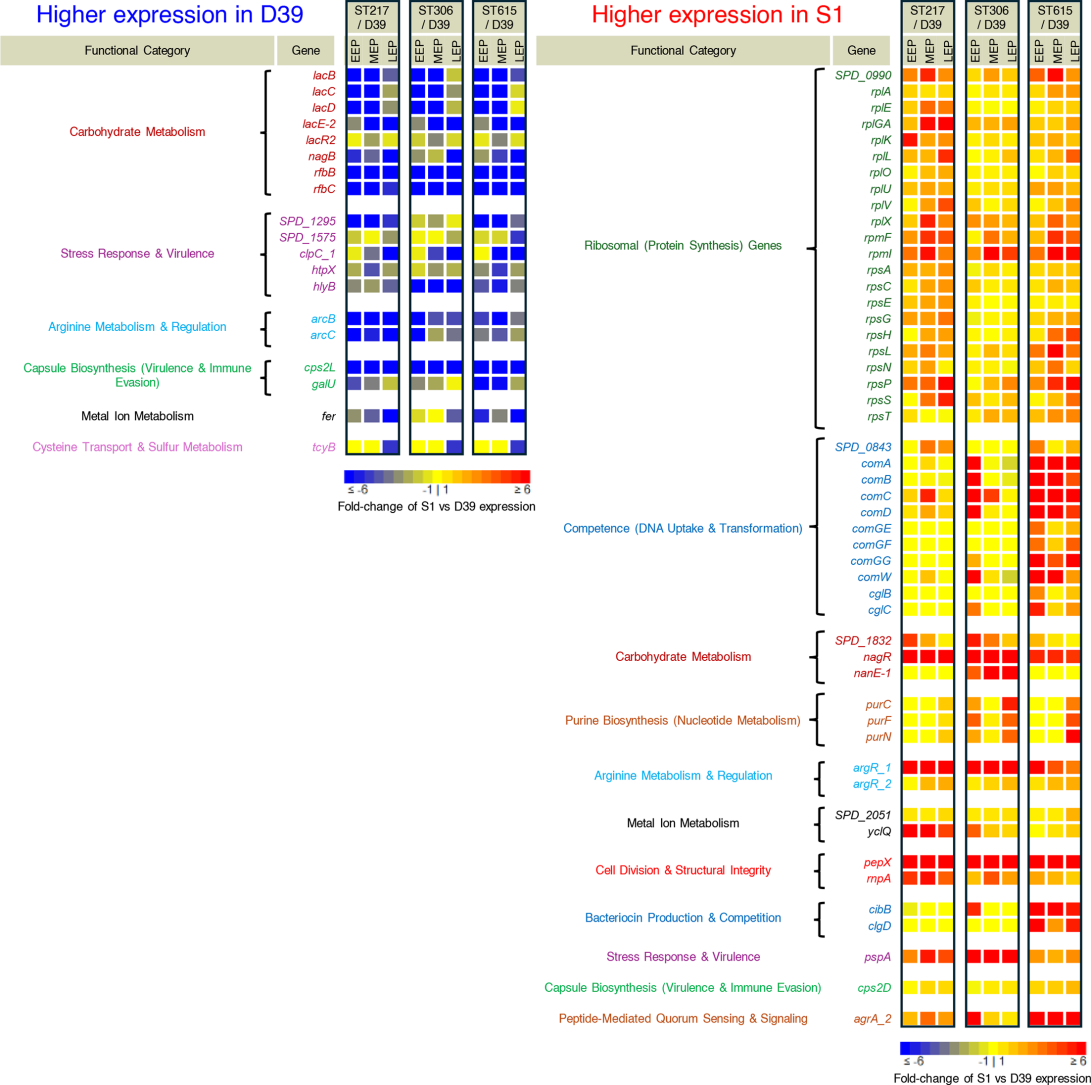
Differential gene expression between pneumococcal serotype 2 (D39) and S1 (ST615, ST615 and ST217). Heatmaps showing DEGs during the exponential growth phase. The left panel displays genes with significantly higher expression in D39 compared with S1 strains, while the right panel shows genes with significantly higher expression in S1 strains compared with D39. DEGs were identified using DESeq2 with thresholds of |log2FC|≥1.0 and adjusted *P*-value <0.05 (Benjamini–Hochberg correction). Expression values are represented as TPM-based fold change, with intensity indicated by the colour bar below each panel. To avoid ratio inflation at low expression levels, TPMs were raised to a minimum value of 10 before calculating the fold changes. Genes more highly expressed in D39 included carbohydrate metabolism and transport systems, particularly the lactose operons (*lacABCD* and *lacTFEG*), amino acid metabolism genes and metal uptake systems. These signatures are consistent with D39’s adaptation to laboratory conditions and its broader utilization of diverse carbon sources. By contrast, S1 strains showed higher expression of genes associated with ribosomal proteins, purine biosynthesis and competence pathways (*comCDE*, *comX* and downstream late competence regulons). These transcriptional signatures suggest that S1 isolates allocate greater resources towards protein synthesis and genetic competence during exponential growth, features that may contribute to their invasive potential.

To investigate the biological significance of these differential expression patterns, all DEGs were categorized into clusters of orthologous genes (COGs) for putative functional assignment. Our results indicated that the most strongly DEGs (whether higher or lower compared to D39) belonged to the carbohydrate transport, competence, purine metabolism and ribosomal/translational functional groups (Fig. 2c). Of the DEGs common to all three pneumococcal S1 strains, the DEGs functionally related to lactose operon I (i.e. genes *lacABCD*) and operon II (i.e. genes *lacTFEG* belonging to the phosphoenolpyruvate phosphotransferase system (PTS)), showed the strongest decrease in expression, ranging from 28.4- to 70.5-fold lower normalized expression compared to D39. Twenty genes associated with functions related to ATP-binding cassette (ABC) transporter were found to be differentially expressed: six of them (SPD_0049, SPD_1652, SPD_0151, SPD_0656, SPD_0917 and SPD_121) showed higher expression levels (ranging from 2.1- to 10.9-fold) and the other 14 genes (SPD_0334, SPD_1621, SPD_0654, SPD_0411, SPD_0742, SPD_0741, SPD_0412, SPD_1466, SPD_0597, SPD_1525, SPD_0555, SPD_0846, SPD_0617 and SPD_0618) had lower expression (ranging from 2 to 43.6-fold) with SPD_0846, SPD_0617 (tcyB) and SPD_0618 (glnM_2), showing the lowest levels at 8.1-, 31.6- and 43.6-fold lower normalized expression compared to D39 (Fig. 3, Table S2).

While glucose is the most preferred carbon source for *Sp*, the presence of several other sugar-specific systems, including the lactose utilization system mentioned above, illustrates the ability of this bacterium to utilize a variety of sugars. Interestingly, *nagR* was found to be the most highly DEGs at 602-fold higher expression in S1 strains compared to D39. The current literature on *nagR* is scarce in relation to *Sp* [34]. Previous authors have documented its role as a repressor of *nagA* and *nagB* [35], which encodes for a *N*-acetylglucosamine-6-phosphate deacetylase and a glucosamine-6-phosphate deaminase, respectively, both needed for the utilization of *N*-acetylglucosamine. NagB catalyses the conversion of d-glucosamine 6-phosphate (GlcN6P) to d-fructose 6-phosphate and ammonia. In line with this, our results show that the higher expression of *nagR* is aligned with the 6.85-fold lower expression of *nagB* in S1 strains compared to D39. The gene *nagA* (SPD_1866) was also found to be 8.3-fold lower in S1 compared to D39 (Table S2), albeit not significant due to high variations amongst the S1 strains.

Sialic acid (or *N*-acetyl neuraminic acid, Nan) represents another important source of carbohydrates for *Sp*. It was also shown to act as a receptor for pneumococcal adhesion and invasion and a trigger signal for the promotion of biofilm formation, with a critical role in nasopharyngeal carriage and invasion of the lungs [36]. The *nanE* (SPD_1497) gene was found to be at 15.3-fold higher expression in S1 compared to D39, with the highest expression levels found in ST306 (45.9-fold). NanE converts ManNAc-6P to GlcNAc-6P and belongs to the *nan* operon I, which consists of ten genes [37]. Activation of the *nan* gene cluster is expected as a specific response to sialic acid, regulated by *nanR* as a transcriptional activator. Our results did not show any significant differential expression in *nanR,* nor any other genes belonging to the *nan* operon I, e.g. *nanP* (SPD_1496), *nanUVW* (SPD_1493, 1494 and 1495), suggesting a unique regulation of *nanE*.

The genes *rfbB* (SPD_0330) and *rfbC* (SPD_0329) were amongst the lowest DEGs in S1 compared to D39, at 33.1-fold and 13.8-fold lower expression. The *rfb* genes were shown to be involved in the dTDP-rhamnose synthesis pathway of *Streptococcus mutans* [38] and *Streptococcus pyogenes* [39]; however, their functions in *Sp* remain to be elucidated [40,41]. A cysteine/methionine ABC transporter encoded by the *tcyB* gene (SPD_0617) [33,34] was also found to be expressed 31.6-fold lower in S1 strains compared to D39. The role of *tcyB* and other Cys/Met ABC transporters in *Sp* remains largely underexplored [42]. A recent study reported that methionine starvation could lead to a survival mechanism, whereby bacterial growth may be inhibited, hence allowing for a prolonged survival [43]. The observed regulation of methionine metabolism genes in S1 strains may be biologically relevant, as methionine availability is limited in the human upper airway [42]. This finding raises the possibility that S1 isolates have adapted strategies to survive in methionine-restricted environments, which could contribute to their epidemiological success. While our data cannot directly establish this link, it provides a basis for future experiments under host-mimicking conditions.

Genes involved in arginine metabolism were among the most DEGs: *argR1* (SPD_0786) showed 16.2-fold higher expression in S1, while *argR2* (SPD_1063) exhibited a 2.5-fold higher level compared to D39. The gene *argR2*, encoding a regulatory protein, was documented to play a key role in maintaining pneumococcal fitness [44,45]. ArgR was previously documented as a repressor of genes such as *argB/F* (SPD_1976, ornithine carbamoyltransferase) and *arcC* (SPD_1977, carbamate kinase). This is aligned with our findings where the *argB/F* and *arcC* genes were expressed at about sixfold lower levels in S1 compared to D39 (Fig. 3, Table S2).

**Fig. 4.**
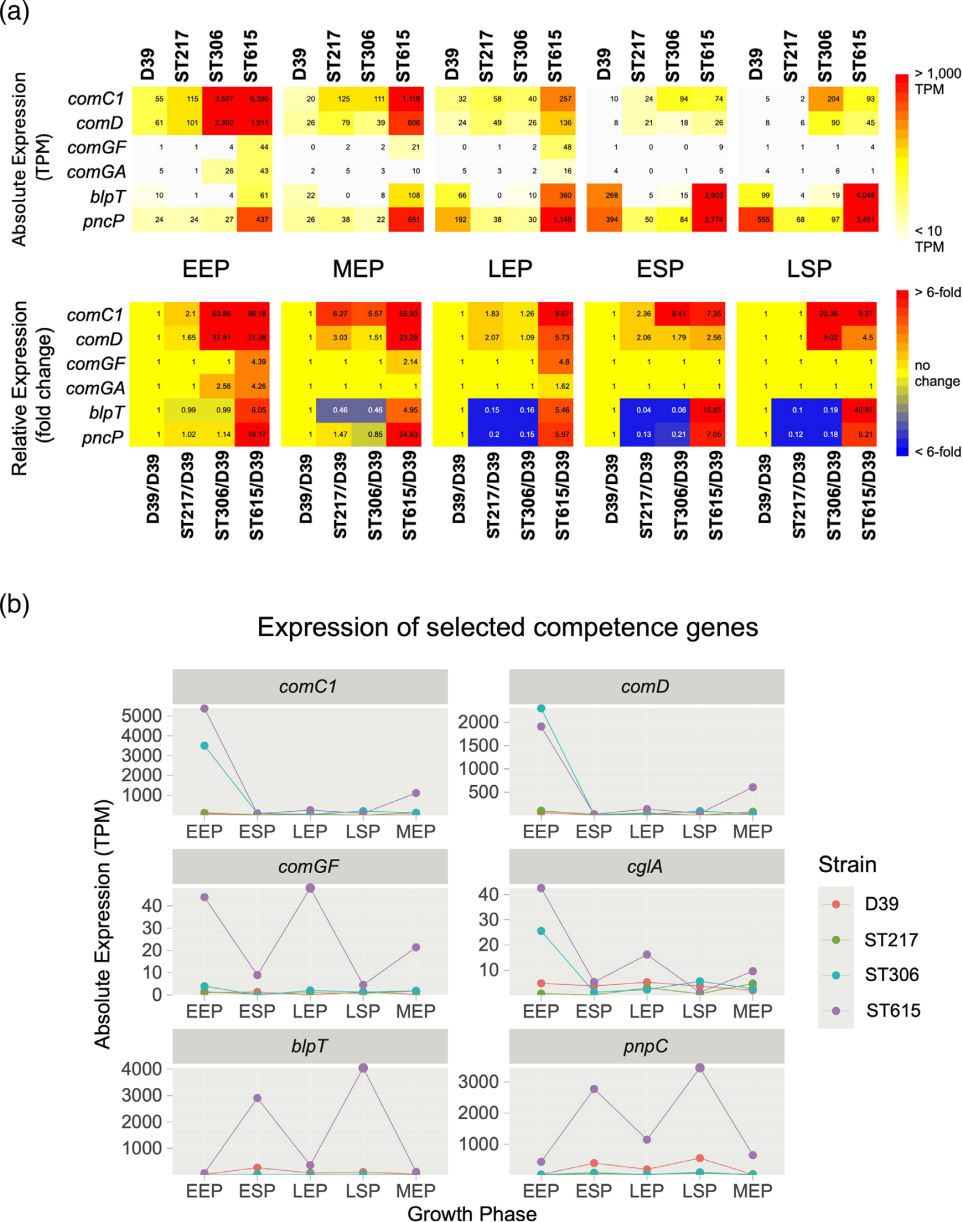
Gene expression of pneumococcal competence-associated genes. (**a**) Heatmaps showing absolute and relative expression values for competence-associated genes, generated using the interactive StrepCom browser (https://bioinf.gen.tcd.ie/cgi-bin/strepcom.pl). The upper panel displays absolute expression values as rounded TPM, while the lower panel displays relative fold changes compared to D39. For fold change calculations, TPM values of lowly expressed genes were adjusted to a minimum of 10 TPM to avoid inflation of ratios. The heatmaps allow direct comparison across S1 strains (ST615, ST217 and ST306) and the reference strain D39, illustrating consistent higher expression of competence pathways in the clinical isolates. (**b**) Line graphs showing expression profiles of representative early (*comC* and *comD1*) and late competence genes (*comGF*, *comGA*, *blpT* and *pncP*) across different growth stages: EEP, MEP, LEP, ESP and LSP. Each profile is plotted separately for ST615/S1, ST217/S1, ST306/S1 and D39/S2, highlighting both conserved and strain-specific patterns of competence gene induction. Clinical S1 strains exhibited stronger and earlier activation of competence regulons compared with D39, consistent with their transcriptional emphasis on genetic competence identified in the broader RNA-seq analysis.

### Competence and bacteriocin pathways define the S1 signature

All strains were profiled with three biological replicates per growth phase, and replicates clustered tightly by strain/phase in the PCA, confirming technical robustness (Fig. 1b; Methods). Differential expression analysis identified competence genes – including *comCDE* and other early/late regulon members – as consistently higher expressed in S1 relative to D39, with fold changes ranging from 8- to 45-fold (Figs3  5a, b; Tables S2 and S3). Other competence-related genes, including *comA*, *comB*, *celA* (SPD_0843), *cglB (SPD_1862)*, *cglC (SPD_1861)* and *cglD (SPD_1860*) competence regulons [46,47], were also found to be significantly highly expressed in S1 strains compared to D39, ranging from 8- to 11-fold higher normalized expression.

We also observed the trio of genes, *purN, purC* and *purF*, involved in the *de novo* purine biosynthesis pathway that is required for pathogenesis [48], were expressed about sixfold higher. Finally, the gene *pepX*, reported previously for its role in the virulence of *Sp* [49]*,* was also found to be expressed about 23-fold higher in S1 strains than in D39 (Fig. 3, Table S2). Of the strain-specific DEGs, no predominant functional groups were observed (Fig. 2c).

**Fig. 5.**
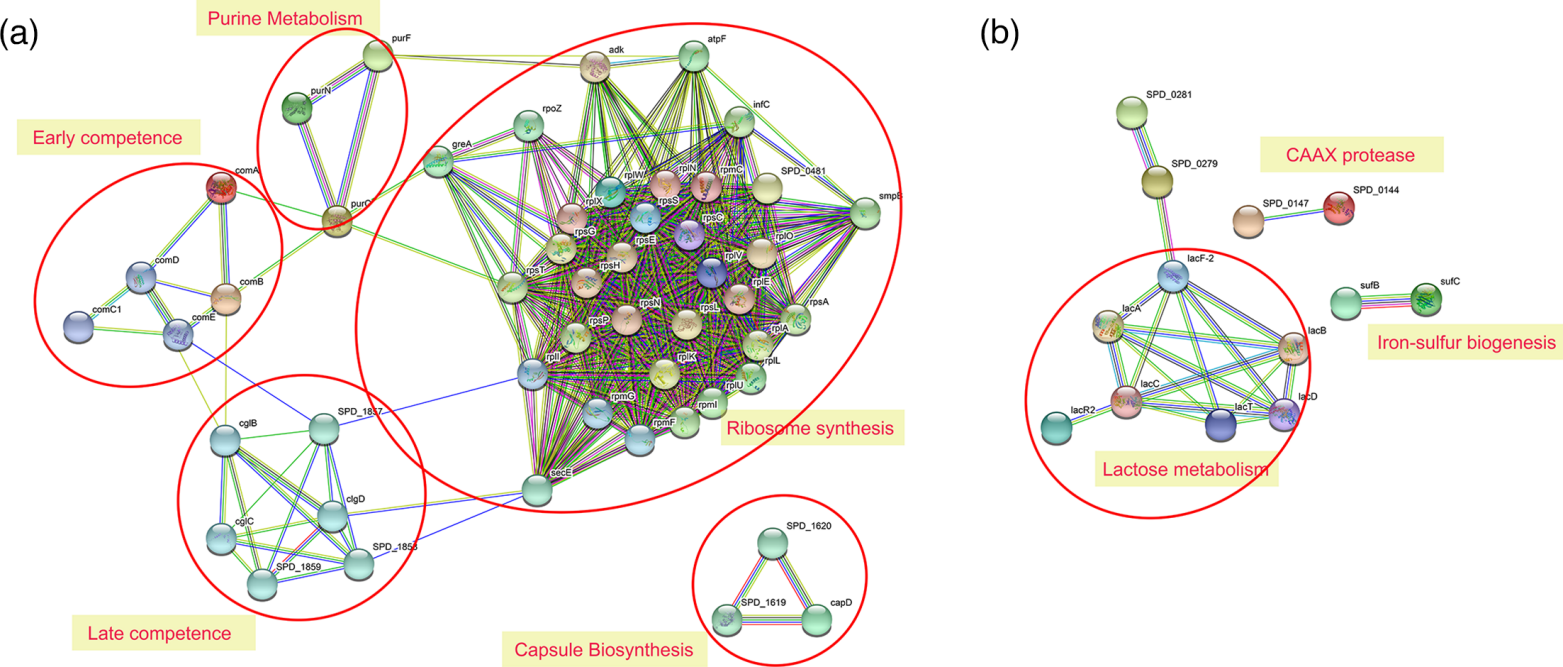
STRING analysis of genes differentially expressed in all three *Sp* S1 strains, compared to D39. STRING protein–protein interaction networks were generated for the subset of DEGs that were consistently higher or lower expressed across all three S1 strains relative to D39. DEGs were identified using DESeq2 with thresholds of |log2FC|≥1.0 and adjusted *P*-value <0.05. Functional enrichment and clustering analyses were performed using the STRING database (version XX; confidence score ≥0.7). Highly connected functional clusters are highlighted by large, shaded circles, which indicate groups of genes with predicted strong associations and biological coherence. (**a**) Genes with higher expression in S1 strains compared with D39 formed clusters related to ribosomal proteins and translation, the competence regulon (including *comCDE* and late competence genes), capsule biosynthesis and purine metabolism. These shaded clusters represent coordinated transcriptional higher expression of pathways involved in protein synthesis, DNA uptake and nucleotide biosynthesis, consistent with the emphasis of S1 strains on rapid growth, genetic competence and capsule production during the exponential phase. (**b**) Genes with lower expression in S1 strains compared with D39 formed shaded clusters associated with lactose metabolism (*lacABCD* and *lacTFEG*), iron–sulphur cluster biogenesis and CAAX proteases. These lower expressed modules suggest a reduced reliance of S1 isolates on certain carbohydrate substrates and metabolic pathways, reflecting divergence from the laboratory-adapted D39 strain. Together, the shaded clusters highlight how conserved gene expression differences between S1 isolates and D39 map onto coherent functional modules that likely underpin their distinct ecological and pathogenic behaviours.

ST615 showed the strongest induction for several competence loci (*comC* FC=91.8, *comD* FC=33.8 and *comGF* FC=90.5), marking it as the upper bound of the S1 expression spectrum. The *blp* bacteriocin cluster was also markedly elevated in ST615 during late phases (6.2- to 41-fold) (Fig. 5b, Table S2).

Together, these results demonstrate that competence activation is a conserved feature across all S1 strains and replicates, while the magnitude of induction varies by lineage, with ST615 displaying accentuated expression of both competence and *blp* regulons. This pattern is consistent with published models in which competence output and competence–bacteriocin coupling vary depending on genetic background and environmental inputs [47,50, 51].

### Protein–protein interaction networks of DEGs in S1 strains

Next, we interrogated the existence of protein–protein interactions or protein networks of the DEGs using the STRING algorithm [52]. Of the DEGs associated with significantly higher expression levels and common to all S1 strains, i.e. ST615, ST217 and ST306, the STRING analysis showed a large network of DEGs encoding strongly associated ribosomal proteins, along with smaller clusters of genes involved in competence and purine metabolism (Fig. 4a) – the patterns were consistent with results shown in Fig. 2(c). Interestingly, genes involved in capsule biosynthesis were also found to be strongly connected in the network, notably *capD (SPD_0099)*, *SPD_1619* and *cps2D (SPD_0318*), the latter of which has an essential role in the synthesis of a mature *Sp* capsule [53]. Of the shared DEGs showing lower expression levels compared to D39, as expected, a strong interaction was observed amongst the DEGs involved in lactose and galactose metabolism (Fig. 4b): of the eight genes belonging to the *lac* operons *lacA-lacB-lacC-lacD* and *lacT-lacF-lacE-lacG*, all of them were found in the three S1 strains. Current literature has documented *lacT* as an activator of the *lacTFEG* operon, while SPD_1044 (*lacR2*) acts as a repressor of the *lacABCD* operon [54]. Interestingly, this repressor lacR2 (SPD_1044) was also significantly lower expressed in S1 compared with D39 (2.1-fold). This is noteworthy because both the repressor (lacR2) and its target operon (lacABCD) show reduced expression, suggesting that the regulation of lactose metabolism in S1 strains may involve additional layers of control beyond lacR2 alone.

### Differential expression of known virulence factors and competence genes

*Sp* is well-known for its ability to induce invasive diseases across all ages, and its virulence mechanisms have been well documented. We cross-referenced our list of S1-shared DEGs against the pneumococcal virulence factor database (VFDB: http://www.mgc.ac.cn/VFs/). Only two of the VFDB pneumococcal genes matched our list of shared DEGs, i.e. SPD_1652 (6.7-fold higher), encoding the iron-binding protein PiuA, and SPD_0888 (3.5-fold lower), encoding the laminin-binding protein Lmb (also known as AdcAII). As mentioned above, the *piuA* gene was expressed at higher levels (6.7-fold) across all three S1 pneumococcal strains. The *lmb/adcAII* gene [55,57] was expressed at statistically significantly lower levels (3.5-fold) in S1 strains. Other genes which have been implicated in colonization, such as *nagA*, *pavB* and *thiD*, were turned on during the LEP (Table S2), although their expression did not reach statistical significance due to large variations. *nagA* has been associated with *N*-acetylglucosamine metabolism relevant for mucosal colonization [35], *pavB* encodes an adhesin known to mediate binding to host extracellular matrix proteins during colonization [58] and *thiD* has been linked to thiamine metabolism pathways that contribute to pneumococcal persistence in the host [59].

Other competence genes were also more highly expressed in S1, especially in ST615, compared to D39 (Fig. 4a, Table S2). These included early competence genes such as *comA*, *comB*, *comC* and *comD,* as well as late competence genes (e.g. *comGF*, *celA*, *cglB* and *cglC*) [47,60]. Competence is known to be a highly regulated process in pneumococci and is dependent on the secretion of the competence-stimulating peptide (CSP), which in turn binds extracellularly to a histidine protein kinase (ComD), leading to the activation of the transcription factor ComE (Fig. S2). ComE activates the expression of the early competence genes, including *pa*, which encodes an alternative sigma factor that drives the expression of late competence genes encoding proteins involved in processes necessary for transformation, such as DNA uptake and homologous recombination (Fig. S2).

Upon permissive conditions, competence is very briefly switched on during the exponential growth phase, with a very short time window allowing for natural transformation [47,61, 62]. Of all the four strains included in the study, ST615 showed the highest expression of competence genes under the given conditions (Table S3), as illustrated by the TPM value plots (Fig. 5a, b) of selected genes representative of early (*comC* (FC=45.6; adjusted *P*-value=0.0013), *comD* (FC=20.6; adjusted *P*-value=0.0009) and late competence genes (*comGA*/*cglA* (SPD_1863) (FC=4.1; expression level too low to pass our threshold for statistical analysis) and *comGF* (SPD_1858) (FC=34.8; adjusted *P*-value=0.0044). For the early competence genes, the expression was high in the early growth phases in strains ST306 and ST615 and then reduced as the bacterial cultures entered LEP, while the expression of the late competence genes remained high until the stationary phase (at least for ST615). Note that *comX* was not included in the original list of DEGs (Table S2), since there are two identical copies of the *comX* genes in the pneumococcal genome [63,64]. This meant that short reads from those transcripts mapped to two locations on the genome and were excluded from the statistical analysis. However, when considering reads from both copies as combined expression values for *comX*, we observe an eightfold increase in expression in S1 compared to D39 (222 TPM vs. 28.1 TPM), but with large variation amongst strains. We also plotted the TPM values for two genes (Fig. 5b) in the bacteriocin (pneumocin) *blp*-regulon (*blpT* (SPD_0466) (FC=2.68 ; adjusted *P*-value=0.531) and *pncP* (SPD_0475) (FC=4.98; adjusted *P*-value=0.209), since regulation of these genes by the dedicated *blp*-regulatory system has been shown to be influenced by the competence system [50]. Interestingly, we noted that the *blp* bacteriocin gene cluster (*blpH*, *blpR*, *blpS, blpT* and *blpY*) was highly expressed (6.2- to 41-fold) during the later growth stages in ST615. The observation that competence genes involved in natural transformation, along with the *blp* regulon, were spontaneously switched on during the planktonic growth of S1/ST615 in BHI suggests that regulatory cues for competence and bacteriocin expression in S1 pneumococci may be highly different from those of D39.

## Discussion

The pneumococcal species includes over 100 distinct capsular serotypes, each showing varied potential to cause IPD [65,68]. S1 is notable amongst these as one of the most virulent, which has prompted further research into its distinctive biology. In previous work, we reviewed S1’s epidemiological characteristics, genomic features and mechanisms of virulence [12]. Building on this foundation, our current study explores the *in vitro* transcriptomic profiles of three distinct lineages of pneumococcal S1, ST306, ST217 and ST615, representing the diverse geographic clusters of Europe, Africa and South America, respectively. The D39 strain was selected as a reference, as S1 has a markedly greater invasive potential compared to D39 in various *in vivo* models [7,8]. Here, we highlight key differences in S1 gene expression and virulence-associated phenotypes, offering valuable resources to the scientific community via the StrepCom platform.

One of our most significant findings was the differential expression of metabolic genes among the S1 *Sp* strains when compared to D39. Specifically, we observed a notably lower expression of the lac gene cluster (*lacABCDTEFG*), arranged in two operons [54], across all S1 strains under our experimental conditions. This cluster is essential for lactose metabolism, comprising genes for lactose uptake (PTS genes *lacEF*), hydrolysis (beta-galactosidase *lacG*) and subsequent metabolic steps. For instance, *lacA* and *lacB* encode the A and B subunits of galactose-6-phosphate isomerase, while *lacC* and *lacD* are involved in tagatose-6-phosphate and tagatose-1,6-diphosphate metabolism, respectively [54]. The reduced expression of these genes in S1 suggests a unique regulation of lactose utilization, potentially influencing energy management and metabolic adaptation in these strains during planktonic growth in BHI medium. This distinct metabolic behaviour could be linked to adaptation of S1 to specific host environments or reflect a preference for other carbon sources in these pathogenic strains. Future studies could explore whether these differences in lactose metabolism play a role in the ability of S1 strains to outcompete other pneumococcal serotypes in the human host, or whether they contribute to resistance to antimicrobial agents targeting metabolic pathways.

Our study also highlights the differential regulation of iron acquisition pathways in S1 strains, consistent with previous reports of their importance in *Sp* survival and pathogenic potential, though further *in vivo* validation will be required to confirm their direct role in virulence. Iron is a vital cofactor for numerous enzymes and cellular processes in bacterial pathogens [69]. Three primary protein complexes, PiaABC, PiuABC and PitABC, serve as iron transporters in *Sp* [70]. Our results indicate a significantly higher expression of the *piu* genes [71] in S1 strains relative to D39 (Table S2). The PiuBCDA iron transport system, particularly the substrate-binding protein PiuA (SPD_1652), facilitates iron acquisition by binding soluble iron–catechol complexes and can also bind haemin and haemoglobin [72]. The enhanced expression of the *piu* genes in S1 strains suggests differences in iron acquisition gene regulation between these strains and D39 under the tested conditions. Previous research has indeed demonstrated that deletion of *piuA* significantly reduces virulence in both *in vivo* and *in vitro* models [73], reinforcing the likelihood that iron acquisition is integral to the pathogenicity of S1. These findings offer a compelling direction for future research, where the role of iron in S1 virulence could be further explored, and ask whether disrupting iron acquisition pathways could provide a therapeutic strategy for combatting S1 infections.

Our study suggests that certain S1 lineages may activate competence genes for natural transformation when cultured in BHI. Notably, we observed markedly higher expression of both early and late competence genes in the ST615 strain compared to D39 and the other S1 strains. Furthermore, ST615 exhibited elevated expression of bacteriocins from the *blp* locus, which is involved in interspecies competition and can contribute to virulence [51,74]. The competence system in *Sp* is known to be influenced by environmental cues, such as pH and antibiotic exposure [75,76], and elevated competence could enhance the strain’s ability to acquire new genetic material, potentially increasing its adaptability and pathogenic potential. We observed higher expression of the late competence gene *cbpD*, encoding a murein hydrolase, which could promote autolysis and the release of pneumolysin. Pneumolysin is a potent virulence factor, and its release via cell lysis could enhance invasiveness [7]. Additionally, competence can increase the display of surface-associated virulence factors through decreased capsule expression, improving adhesion to host tissues and further contributing to pathogenicity [77,78]. Taken together, these findings point to the possibility that pneumococcal S1 strains, particularly ST615, may exhibit competence-associated functions that enhance virulence and adaptability in host environments [21,79]. This raises an intriguing hypothesis on the impact of horizontal gene transfer in the evolution of pneumococcal S1 virulence. Similarly, our transcriptomic analysis uncovered significant changes in the regulation of ribosomal protein genes, with S1 strains displaying higher expression of genes encoding ribosomal proteins from both the 50S and 30S subunits compared to D39. These findings indicate that S1 strains show transcriptional signatures with higher expression of protein synthetic genes than strain D39.

While our data do not directly address proteome dynamics, previous studies have shown that variation in ribosomal and stress-response gene expression can influence pneumococcal adaptability to changing environments [16,47]. Given that increased translation rates can enhance the overall output of protein synthesis, this could provide S1 strains with greater capacity to adapt to environmental stresses. Our study does not directly test virulence; however, prior work has shown that elevated ribosomal activity and translational capacity are associated with bacterial fitness and survival during infection [47,80].

The transcriptional signatures may help explain several well-documented features of S1 strains. For instance, the S1 increased expression of competence and bacteriocin systems, particularly in ST615, may facilitate rapid adaptation and intra-host competition, consistent with S1’s tendency to cause outbreaks [81]. Similarly, lower expression of lactose metabolism and alternative carbohydrate pathways mirrors previous observations that S1 isolates are less adapted for asymptomatic colonization, which may partly account for their low prevalence in carriage studies [10,66, 82]. Finally, the emphasis on purine biosynthesis and protein synthesis pathways may reflect prioritization of rapid growth during invasive disease, aligning with epidemiological evidence that S1 is amongst the leading causes of pneumococcal meningitis and bacteraemia in Africa and elsewhere [82,84]. By integrating our transcriptomic findings with these well-established characteristics, our data suggest a molecular basis for understanding how S1 differs from other pneumococcal lineages in both biology and clinical impact.

While we acknowledge the limitation of using BHI medium, which does not reflect the host milieu, several of the transcriptional signatures we observed – particularly for competence and iron uptake – are also engaged *in vivo*, though modulated by host-specific factors. For competence, recent *in vivo* transcriptomic studies during pneumonia and sepsis murine models have shown activation of *com* regulons, though their dynamics differ from those observed *in vitro*, highlighting the influence of host-derived cues such as stress and nutrient restriction [20,21]. For instance, host airway proteases have been reported to degrade CSP, thereby attenuating competence signalling in the respiratory tract [85]. Consistent with this, we found robust expression of the *blp* locus in ST615, which has been directly implicated in within-host bacterial competition and lung pathology in murine pneumonia models [86].

For iron acquisition, the observed expression increase of *piuA* aligns with the concept of nutritional immunity, whereby the host restricts access to essential metals to limit bacterial growth. Haemoglobin, an abundant host iron source, has been shown to remodel pneumococcal growth and transcriptional profiles [17]. In human infection, antibodies against PiuA and PiaA have been detected in convalescent sera from patients with pneumococcal septicaemia, providing evidence that these transporters are expressed and immunogenic *in vivo* [87,88]. Direct bacterial RNA-seq from human CSF has further confirmed robust expression of transporter and stress-response pathways during meningitis [22]. Previously, *ex vivo* studies using human plasma, pleural fluid and CSF have also demonstrated rapid remodelling of metal uptake and metabolic pathways in pneumococcus [23,89]. Our dataset also complements transcriptomic studies performed with clinical isolates or host-proximal conditions. In particular, Ramos-Sevillano *et al*. [19] mapped how human serum and CSF reshape pneumococcal transcription and metabolism across strain backgrounds. Taken altogether, these findings suggest that the transcriptional differences we observed in BHI – especially in competence and iron uptake – reflect pathways that are biologically relevant *in vivo*. As a translational step forward, future work will evaluate these same S1 strains under host-mimicking conditions (iron- or manganese-limited media, in the presence of haemoglobin, or during epithelial co-culture) and benchmark these data against published *in vivo* datasets to clarify which experimental systems most accurately recapitulate host transcriptional states and guide follow-up mechanistic work.

Another limitation of our study is that all comparisons were made against the laboratory strain D39, which is commonly used as a reference in pneumococcal research. It therefore remains possible that some of the observed transcriptional differences – such as the downregulation of lactose metabolism genes – may reflect atypical expression in D39 rather than a consistent pattern unique to S1 strains. Future comparative studies including additional reference strains, such as TIGR4 or clinical isolates of other serotypes, will be necessary to distinguish lineage-specific features of S1 from strain-specific regulatory idiosyncrasies. Exponential-phase samples were analysed together, rather than subdivided into ESP, MEP and LEP contrasts. Our rationale for this approach was to emphasize the core transcriptional programme of active proliferation, which aligns most closely with pneumococcal invasion biology. The PCA confirmed that strain identity, rather than growth phase, was the dominant driver of transcriptome variation (Fig. 1b). Nevertheless, we recognize that phase-specific comparisons (e.g. early vs. late exponential) may reveal additional nuances and represent an important avenue for future investigation.

By presenting our findings in an accessible and open format (https://bioinf.gen.tcd.ie/cgi-bin/strepcom.pl), we aim to encourage follow-up studies and inspire new hypotheses regarding the unique features of S1. The recently updated PneumoBrowse 2 platform [90] provides an integrated genome annotation and transcriptomic visualization tool for *Sp*, complementing our creation of StrepCom by enabling cross-strain comparisons and curated reference data. While PneumoBrowse 2 offers a comprehensive resource for the laboratory strains D39 and TIGR4, our StrepCom browser uniquely extends accessibility to clinical S1 isolates, thereby filling an important gap for researchers studying hypervirulent lineages [83]. While we acknowledge that the clinical relevance of our study may be limited due to the reliance on laboratory-based conditions, the insights into gene regulation, metabolic adaptations, iron acquisition, competence and ribosomal protein expression provide robust starting points for further research. Altogether, our study not only contributes to a deeper understanding of the pathogenesis of S1 but also facilitates the identification of potential therapeutic targets and biomarkers for S1-related diseases.

## Supplementary material

10.1099/mgen.0.001582 Uncited Supplementary Material 1.

10.1099/mgen.0.001582 Uncited Supplementary Material 2.

## Abbreviations

BHI: Brain Heart Infusion
COGs: Clusters of Orthologous Groups
CSF: cerebrospinal fluid
CSP: competence-stimulating peptide
DEG: differentially expressed gene
EEP: early exponential phase
ESP: early stationary phase
IPD: invasive pneumococcal disease
LEP: late exponential phase
log2FC: log2 fold change
LSP: late stationary phase
MEP: mid-exponential phase
PCA: principal component analysis
PTS: phosphoenolpyruvate phosphotransferase system
RNA-seq: RNA-sequencing
S1: serotype 1
*Sp*: *Streptococcus pneumoniae*
ST: sequence type
TPM: transcript count per million
VFDB: virulence factor database
