# Supplementary material for: Transcriptomic analysis of Streptococcus pneumoniae serotype 1 reveals serotype-specific gene regulation
Source: Microb Genom. 2025 Dec 5;11(12):001582. doi: 10.1099/mgen.0.001582 (PMC12690605; doi:10.1099/mgen.0.001582)
Supplement: Uncited Supplementary Material 1. [file mgen-11-01582-s001.pdf]

Transcriptomic analysis of the planktonic growth of *Streptococcus pneumoniae* serotype 1 reveals serotype-specific gene regulation.

Pisut Pongchaikul[1,2,3ϕ]*, Karsten Hokamp[4ϕ]*, Morten Kjos[5], Chrispin Chaguza[6], Stavros Panagiotou[7], Reham Yahya[3], Laura Bricio-Moreno[8], Teerawit Audshasai[3,9], Disa L. Hammarlöf[10], Jay C.D. Hinton[3], Marie O'Brien[3,11], Aras Kadioglu[3].

[1]Ramathibodi Medical School, Faculty of Medicine Ramathibodi Hospital, Mahidol University, Bang Phli, Samut Prakan 10540, Thailand.
[2]Integrative Computational BioScience Center, Mahidol University, Nakhon Pathom 73170, Thailand.
[3]Department of Clinical Infection Microbiology and Immunology, Institute of Infection Veterinary and Ecological Sciences, University of Liverpool, U.K.
[4]Department of Genetics, School of Genetics and Microbiology, Smurfit Institute of Genetics, Trinity College, Dublin, Ireland
[5]Faculty of Chemistry, Biotechnology and Food Science, Norwegian University of Life Sciences, Ås, Norway.
[6] Department of Host-Microbe Interactions, St Jude Children's Research Hospital, Memphis, Tennessee, USA
[7]University of Manchester, Manchester, U.K.
[8]Center for Immunology and Inflammatory Diseases, Massachusetts General Hospital and Harvard Medical School, Boston, MA 02114, U.S.A.
[9]Department of Microbiology, Faculty of Pharmacy, Mahidol University, Bangkok, Thailand.
[10]Department of Cell and Molecular Biology, Uppsala University, Uppsala, Sweden
[11]ReNewVax Ltd, Liverpool, U.K.
[ϕ] Co-first authors
*Correspondence should be addressed to: pisut.pon@mahidol.ac.th, kahokamp@tcd.ie

SUPPLEMENTAL FIGURES & TABLES

Suppl Figure S1

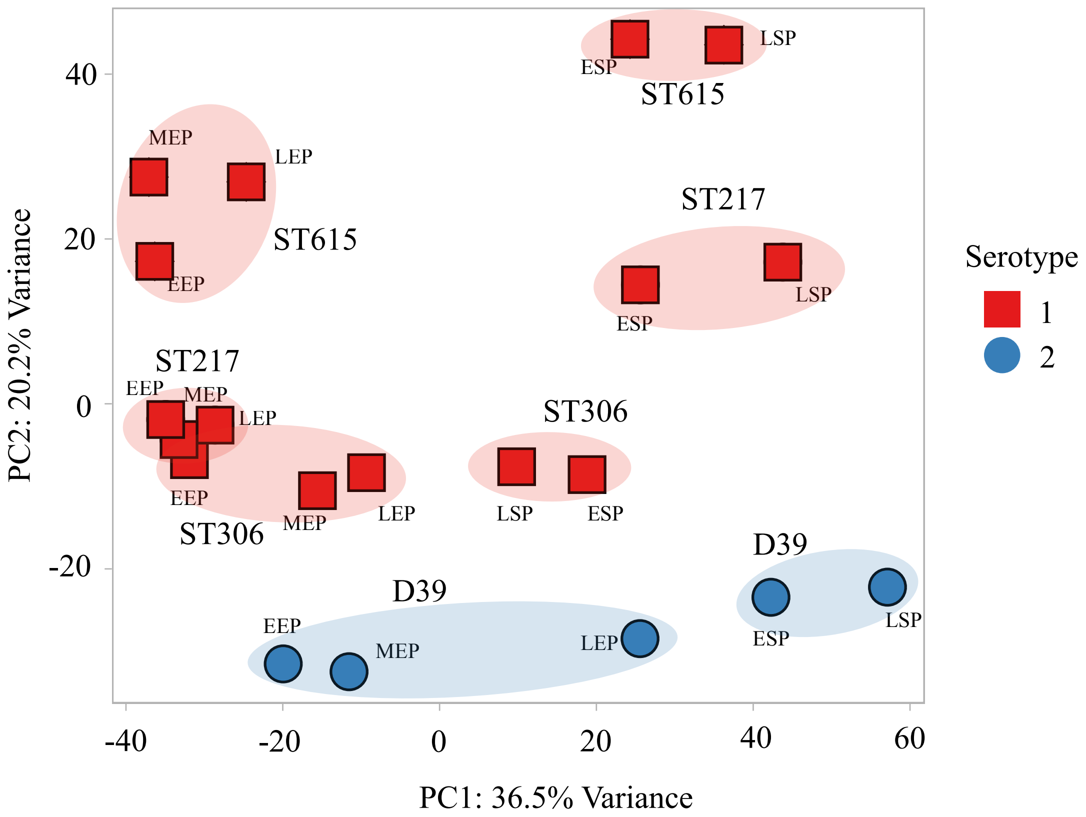

Supplementary Figure 1. Principal component analysis (PCA) illustrating the clustering of the transcriptomic profiles of four *S. pneumoniae* strains obtained from different segments of their respective growth curves.

Abbreviations. EEP: early exponential phase; MEP: mid-exponential phase; LEP: late exponential phase; ESP: early stationary phase; LSP: late stationary phase; ST: sequence type.

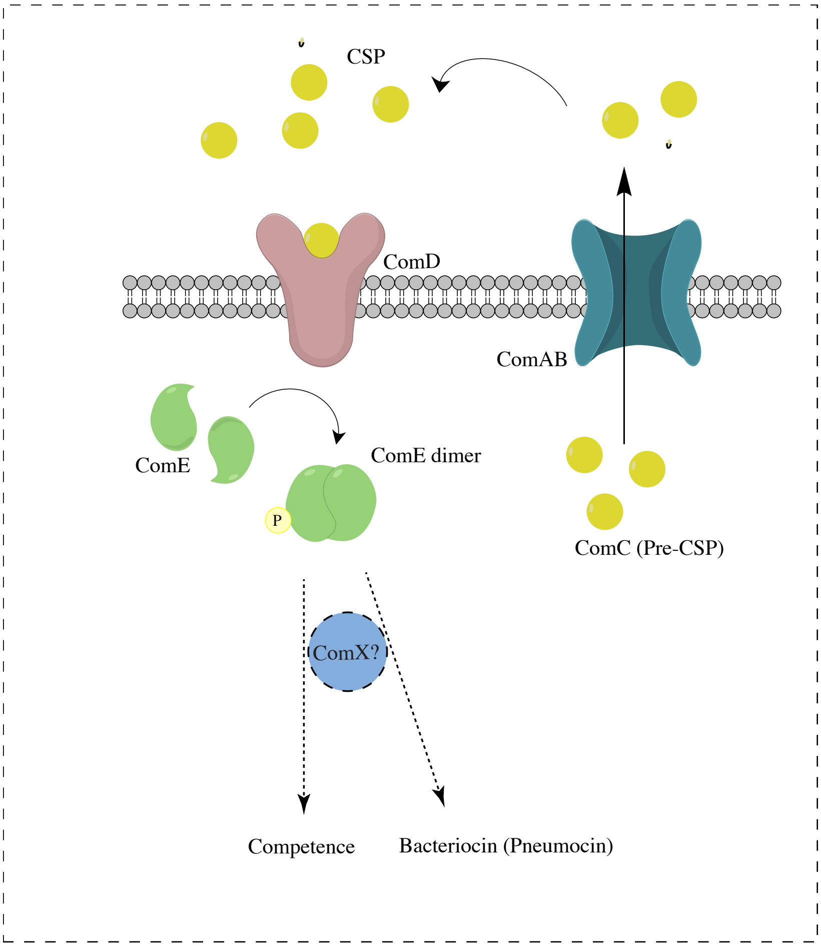

Supplementary Figure 2 Schematic showing the regulatory network regulating competence in *S. pneumoniae*. The ComC precursor of the competence-stimulating peptide CSP, encoded by the *comC* gene, is cleaved and exported out of the cell by an ABC transporter (ComAB). CSP accumulates in the extracellular environment as the cell density increases. Once the concentration of CSP reaches a threshold, the peptide can effectively bind to a transmembrane histidine kinase receptor called ComD, which in turn activates its cognate transcription factor ComE through phosphorylation. ComE promotes expression of early competence genes, the sigma-factor ComX, which is responsible for the activation of late competence genes encoding proteins involved in DNA uptake and integration into the *S. pneumoniae* genome. Expression of the Blp bacteriocin (pneumocin) regulon (*blpT* (SPD_0466) and *pncP* (SPD_0475), have been shown to be influenced by the competence regulatory system.

Supplementary Table 1 (xls file): Summary of RNA sequencing in four strains of *Streptococcus pneumoniae* obtained from five different segments of planktonic growth phases.

Supplementary Table 2 (xls file): List of differentially expressed genes. The list of DEGs obtained from three serotype 1 strains (ST615, ST207 and ST306) compared to that of strain D39 during their exponential phase in BHI broth

Supplementary Table 3 (xls file). TPM of each gene in each isolate. The TPM of each gene obtained from all four isolates from each point of the growth phase.
